# Stem Cells in the Treatment of Insulin-Dependent Diabetes Mellitus

**Published:** 2016

**Authors:** M. A. Borisov, O. S. Petrakova, I. G. Gvazava, E. N. Kalistratova, A. V. Vasiliev

**Affiliations:** Pirogov Russian National Research Medical University, Ostrovitianov str. 1, Moscow, 117997, Russia; Lomonosov Moscow State University, Faculty of Biology, Leninskie Gory 1, bld. 12, Moscow, 119991 , Russia; Koltsov Institute of Developmental Biology, Russian Academy of Sciences, Vavilova str. 26, Moscow, 119334, Russia

**Keywords:** cellular therapy, diabetes, differentiation, gene expression, pancreas

## Abstract

Diabetes affects over 350 million people worldwide, with the figure projected to rise to nearly 500 million over the next 20 years, according to the World Health Organization. Insulin-dependent diabetes mellitus (type 1 diabetes) is an endocrine disorder caused by an autoimmune reaction that destroys insulin-producing β-cells in the pancreas, which leads to insulin deficiency. Administration of exogenous insulin remains at the moment the treatment mainstay. This approach helps to regulate blood glucose levels and significantly increases the life expectancy of patients. However, type 1 diabetes is accompanied by long-term complications associated with the systemic nature of the disease and metabolic abnormalities having a profound impact on health. Of greater impact would be a therapeutic approach which would overcome these limitations by better control of blood glucose levels and prevention of acute and chronic complications. The current efforts in the field of regenerative medicine are aimed at finding such an approach. In this review, we discuss the time-honored technique of donor islets of Langerhans transplantation. We also focus on the use of pluripotent stem and committed cells and cellular reprogramming. The molecular mechanisms of pancreatic differentiation are highlighted. Much attention is devoted to the methods of grafts delivery and to the materials used during its creation.

## MOLECULAR MECHANISMS OF PANCREATIC DIFFERENTIATION

To gain insights into the pancreatic differentiation of cells *in vitro, *the primary stages of pancreatic organogenesis need to be clarified. Numerous studies in mouse models have greatly advanced our understanding of developmental mechanisms and delineated the stages of organ formation (*Table*).

The pancreas plays a crucial metabolic role by producing various hormones and enzymes. The pancreas contains exocrine and endocrine cells. The exocrine compartment consists of acinar cells that produce digestive enzymes, such as amylases, lipases, proteases, and nucleases. These enzymes are released into ducts which form the branching duct system lined with epithelial cells [1]. The endocrine pancreas is composed of cell clusters called islets of Langerhans (iL). Each pancreatic islet is composed of α, β, δ, ε and PP cells that produce glucagon, insulin, somatostatin, ghrelin, and pancreatic polypeptide, respectively.

The pancreas is of endodermal origin. Following formation, the endoderm differentiates into the embryonic gut tube that undergoes regional specification in response to molecular contexts. The pancreas develops as dorsal and ventral buds from the foregut between the duodenum and the stomach [2, 3]. The dorsal bud receives signals from the notochord and dorsal aorta [4], whereas the ventral bud receives signals from the overlying cardiac mesenchyme and the lateral plate mesoderm [5].

**Table 1:** Progression of human pancreas development in vivo [6, 7]

Stage	Stem cell	Endoderm	Embryonic gut	Pancreatic endoderm	Precursor cell	β-cells
Embryonic day	6	14	21-28	30-33	45+	55+
Markers	Oct4^+^ Sox2^+^	Sox17^+^ Foxa2^+^ EpCAM^+^ Sox7^-^ Pdgfra^-^	Sox17^+^ Foxa2^+^	Pdx1^+^ Nkx6.1^+^ Ptf1a^+^ Ki67^+^ Sox9^+^ Sox17^-^	Ngn3^+^ Pdx1^+^ Nkx6.1^+^ Ptf1a^-^	Ins^+^ Nkx6.1^+^ Mafa^+^ Gcg^-^ Sox9^-^

However, fibroblast growth factors (FGFs) and bone morphogenetic proteins (BMPs) exposed to endodermal explants can redirect the fate of pancreatic cells to hepatic lineage. On the other hand, downregulation of the p300-dependent histone acetylation associated with gene expression reverses the hepatic phenotype [8]. On embryonic day E11.5, the mouse ventral and dorsal buds increase in size and merge into a single organ [9]. Along with this fusion, proliferation of pancreatic epithelial cells is mainly guided by the growth factors of mesenchymal cells (E12.5 in the mouse) [10]. Further proliferation promotes branching of epithelial ducts. In parallel with these processes, precursor endocrine cells detach from the epithelium and finally associate into iL. At E16.5, monohormonal insulin-positive and acinar cells arise [11]. The adult human pancreas contains 1 mln iL [12]. During the endocrine differentiation, the islet progenitor cells co-express various hormones, eventually maturing into monohormonal cells [13, 14]. In a mouse model, it was shown that glucagonsecreting cells are the first endocrine cells to occur, being detectable as early as E9.5 [15, 16]. This is followed by the formation of cells co-expressing insulin and glucagon, whereas first insulin-secreting β-cells and glucagon-secreting α-cells are observed from day 14. By E18, somatostatin-producing δ-cells and pancreatic polypeptide-producing PP cells can be detected in the islets [14, 15].

All endocrine cells originate from Pdx1-positive pancreatic progenitors. During pancreas development, Pdx1 is expressed in endocrine and exocrine progenitor cells; however, to the end of specification, Pdx1 expression is restricted to β- and δ-cells [16]. The endocrine cell fate determination is regulated by the transcription factor Ngn3. Its inhibition at E11.5 dramatically suppresses endocrine differentiation [17].

The use of genetic tools has improved our understanding of the transcription factors functions in the generation of different types of pancreatic endocrine cells. These factors include such markers as Sox9, Pdx1, Ngn3, Ia-1, Pax4, Arx, Nkx2.2, Nkx6.1, Nkx6.2, Pax6, and Mafa.

Sox9 is expressed in Pdx1^+^ epithelial cells from E9. At E14.5, Sox9 expression is restricted to uncommitted cells with low Pdx1 expression and not observed in hormone-secreting cells. In postnatal mice, Sox9 localizes in centroacinar cells and certain ductal epithelial cells [19]. There is evidence that Sox9 acts as a marker of pancreatic progenitor cells: its expression remains unaltered in *Ngn3- *and *Nkx6.1-*knockout mice. Transgenic mice with pancreatic precursor cells artificially maintained in the progenitor state demonstrate abnormally constant levels of Sox9 expression. Sox9 and the proendocrine transcription factor Ngn3 are coexpressed on embryonic day 15.5; however, they are not detected in Nkx2.2- and Isl1-positive cells found in mature iL. Deletion of Sox9 in Pdx1^+^ progenitor cells reduces the number of endocrine cells with premature cell differentiation into glucagon- and Isl1-expressing cells. Thus, Sox9 is a marker of progenitor cells and its activity is required to maintain them in a proliferative state and prevent their premature differentiation [20, 21].

Both Sox9 and Pdx1 are co-expressed at E8.5 in the dorsal and ventral endoderm beneath the stomach and duodenum. Later, Pdx1 expression is confined to β-cells, regulating glucose-dependent insulin secretion [22-24]. There are studies that suggest that mature pancreatic cells derive from Pdx1^+^ progenitor cells [25]. This agrees with the pancreatic agenesis in Pdx1-deficient mice [26]. Pdx1 inactivation at different stages of development and in mature β-cells revealed its necessity for the establishment and maintenance of the phenotype of β-cells [27, 28]. Furthermore, Gannon *et al *[29] demonstrated that down-regulation of Pdx1 in β-cells at late stages of embryonic development leads to a decrease in the proliferative capacity of insulinsecreting cells, along with an increased proliferative activity in glucagon-producing cells. These findings support the view that Pdx1 plays an essential role in the specification and differentiation of β-cells, as well as in maintaining the pool of endocrine cells at late stages of embryonic development [29].

In contrast to Pdx1, Ngn3 affects only the differentiation of endocrine tissue. It can be detected from E8.5 with peak expression at E15.5, resulting in a low expression level in mature endocrine tissue. Ngn3 is crucial for all enteroendocrine and endocrine lineage specification [25, 30, 31]. Ngn3 inactivation in mature Pdx1^+^ cells impairs the functions of iL [32], whereas its upregulation induces endocrine cell differentiation [33, 34]. Ectopic Ngn3 expression in Pdx1^+^ cells prematurely converts cells into endocrine lineage, which only produces glucagon [35, 36]. Villasenor *et al *[37] report that developmental Ngn3 expression occurs in two distinct temporal waves that are consistent with the “first” and “second” transitions previously described by Pictet *et al *[38], giving rise to early- and late-forming endocrine cells with different developmental potentials [37, 38]. A study by Johansson *et al *[39] showed that early Ngn3^+^ cells differentiate into α-cells. Activation of Ngn3 at late stages induces the lineage commitments of β- and PP-cells after embryonic day 11.5 and δ-cells after embryonic day 14.5, whereas the emergence of α-cells progressively decreased [39].

The transcription factor Ia-1 is a target of Ngn3 and participates in endocrine cell differentiation. In the case of Ia-1 mutations, endocrine cells are observed but most of them do not secrete hormones [40]. Unlike Ngn3, ectopic expression of Ia-1 in ductal cells is insufficient to induce endocrine differentiation. However, co-expression of Ngn3 and Ia-1 significantly improves endocrine induction efficiency as compared to Ngn3 alone expression [41].

**Fig. 1:**
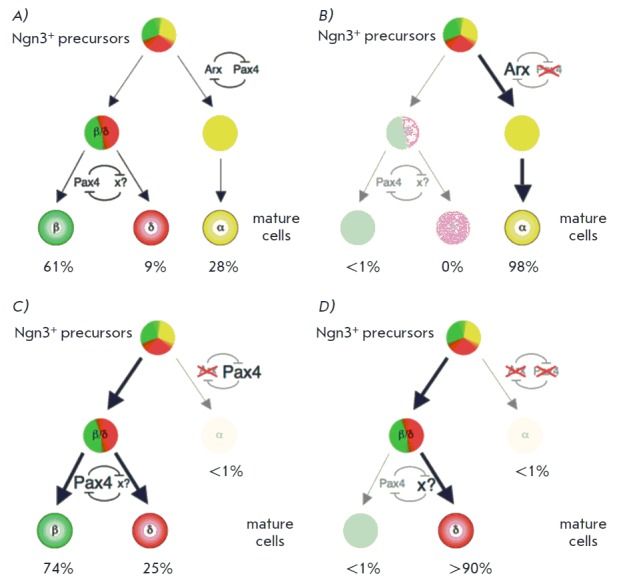
Schematic representation of endocrine cell fate specification during pancreatic development. A) An uncommitted endocrine progenitor cell can become an α-cell or transform into a second progenitor cell with the β- or δ-cell lineage fates expressing Arx and Pax4. B) A change in the cell fate decision is caused by the lack of Pax4; C) A change in the cell fate decision is caused by the lack of Arx; D) A change in the cell fate decision is caused by the lack of Pax4 and Arx [By 18]

Arx and Pax4 play crucial roles in the specification of endocrine cells subtypes. Arx acts as a differentiation promoter of α- and PP-cells, while Pax4 specifies the β- and δ-lineages (*Fig. 1*). Pax4 deficiency fails to promote the differentiation of β- and δ- progenitors into their programmed pathways, leading to an increase in the population of α-cells [42]. On the other hand, Arx loss leads to an increase in β- and δ-cells, with the disappearance of α-populations [43]. Closer analysis showed that the Arx and Pax4 factors act as antagonists. Simultaneous Pax4 and Arx knockout leads to the disappearance of β- and α-cell populations with an increase in δ-cells concomitantly with no changes in the number of PP-cells [44]. The authors conclude that Pax4 is not necessary for the fate of β-/δ-cell determination but inhibits α-cells differentiation by Arx suppression.

At early stages of embryonic development, Nkx2.2 is important for the specification of the β-cells. However, in mature iL, Nkx2.2 appears as a α-, β- and PP-cells marker. Nkx2.2-deficient mice display a loss of α-cells as well as a reduced number of β- and PP-cells, whereas the number of δ-cells remains unaltered [45, 46].

Another marker of pancreatic epithelial cells, Nkx6.1, is observed as early as E9.5. It is first detected in Ngn3+ endocrine progenitors, followed by mature β-cells, in which it regulates insulin secretion [47, 48]. Nkx6.1-knockout mice mostly lack mature β-cells despite the normal development of other islet cell types [47]. Nkx6.2, a paralog of Nkx6.1, shares expression patterns with Nkx6.1, but it is not observed in mature β-cells [49, 50]. Nkx6.2-knockout mice exhibit a wildtype phenotype, whereas Nkx6.1/Nkx6.2 double-mutant mice show phenotypic alterations characteristic of a Nkx6.1 mutant phenotype, concomitantly with a striking decline in glucagon-producing cells. Based on these findings, the authors suggest a broader role for Nkx-factors in α-cell specification [49].

Another member of the Pax family, Pax6, is critical for the differentiation of islet cells. Pax6 is expressed in all endocrine hormone-producing cells. Pax6 guides the differentiation of the four islet cell types and iL development, as evidenced by Pax6 loss in mice [51, 52].

Members of the Maf gene family (Mafa*, *Mafb and cMaf) control the terminal differentiation of β- and α-cells. Mafa binds to the promoter in the insulin gene and acts as a strong transactivator of insulin gene expression [53-55]. Mafa gene expression is induced at E13.5 and confined to just insulin+ cells during embryonic development and after birth [56]. Mice lacking Mafa have diabetes type 1 with a pronounced decrease in insulin blood levels and perturbed islet organization. Mafa deficiency abrogates glucose-dependent secretion of insulin in isolated insulin+ cells [57]. In addition, ectopic Mafa expression in the endoderm of chicken embryos and in cell cultures of nonpancreatic cells is sufficient to trigger insulin production [58].

Overall, the description of the key genes’ roles in the specification of different endocrine cells unveils the complexity of their regulation mechanisms. The objectives for the next few years are *in vitro *and *in vivo* studies of insulin-producing β-cells genesis for the optimization of *in vitro *cell differentiation programs.

## TRANSPLANTATION OF DONOR ISLETS OF LANGERHANS

Transplantation of a pancreas is a promising therapy for patients with diabetes [59]. However, this approach contains procedural risks for the recipient and leads to the need for lifelong immunosuppression.

Transplantation of allogeneic isolated islet cells allows one to avoide abdominal surgery. In 1983, human iL were transplanted to rats with experimentally induced diabetes [60]. The first allogenic iL transplantation into a Type I diabetic patient was reported in 1990 [61]. However, the efficiency of this approach remained extremely poor until 2000. It is likely that this was due to the limited techniques of islet isolation available at the time, low islet yield, and severe immunosupression. Shapiro *et al *[62] developed the Edmonton protocol that reduced the alloimmune response and improved the survival rate of transplanted islets [62-64]. Owing to this protocol, the need for exogenous insulin was eliminated following islet transplantation. Moreover, Shamoon *et al *[65] reported that in patients receiving therapy glycosylated hemoglobin HbA1c reached a normal level. The Edmonton protocol employs an enzymatic dissociation of islet cells. Islets are infused intraportally by portal vein catheterization, after which the cells become trapped in the venous sinuses of the recipient, have access to oxygen supply, and initiate glucose- dependent insulin secretion. The important step in this procedure is a combination of immunosuppressive agents. Following infusion, the recipient receives daclizumab to prevent initial rejection. The use of another immunosuppressive component, sirolimus, allows one to avoid corticosteroid use, which shows toxicity to islet cells. The third agent, tacrolimus, is administered at small doses to minimize the side-effects it has on the islet mass. Current immunosuppressive therapies are successful at reducing graft rejection rates and prolonging islet survival up to 5 years [66-68]. However, the risks of long-term immunosuppression, as well as profound shortage of donor material, hinder the widescale application of this procedure.

The similarity of human and porcine insulin [69, 70] and successful use of porcine insulin in diabetic patients before the recombinant human insulin was first produced [71] allowed researchers to consider porcine islet cells as material for transplantation. Encapsulation procedures have been created to ameliorate rejection responses. A recent study from Living Cell Technologies showed that encapsulated porcine islets are safe and potent without the need for immunosuppressive agents when used in diabetes type 1 patients (http://www.lctglobal.com). Neither inflammation nor subsequent fibrosis, nor increase in glycosylated hemoglobin levels, was observed following a progressive reduction in daily insulin doses [72-74].

There are a number of studies that have achieved a positive outcome with encapsulated human islets [73, 75-78]. However, despite the absence or reduced need for immunomodulation, the limited availability of donors remains the major limiting factor.

Encapsulation materials may include water-soluble (alginate hydrogels) and water-insoluble polymers [79]. Although alginates are water-soluble, they remain intact over several years [78, 80-84]. Creating doublelayered capsules contributes to decreased membrane porosity and enhances membrane durability and better immunoisolation. For protection against immune destruction, membranes can be coated with poly-L-lysine and polyornithine in prejudice of mechanical stability and durability [79, 85, 86].

Alternative sites for grafting have remained the focus of numerous studies. To attenuate early graft loss and to yield an insulin delivery environment, implanted islets need to be vascularized for appropriate oxygen and nutrient supply. Unfortunately, current research efforts have not been very successful with an ideal site for encapsulated islet transplants due to their sizes. Appropriate for the transplantation of a non-encapsulated pancreatic islet, grafts sites (such as the liver and spleen) are space-limited to accommodate a large capsule volume (diameter ranges from 600 μm). Common laparoscopic techniques allow one to implant a capsule into the abdominal cavity. However, abdominal mesothelial cells trigger a severe immune response indirectly through macrophages and directly by producing TNF-α, IL-1β, and IL -10, and other cytokines [87]. Better outcomes were observed both with encapsulated and non-encapsulated islets when implanted beneath the renal capsule or subcutaneously [88]. These sites yielded a mild cytotoxic response, concomitantly with high islet survival rates and graft function [89].

## THE USE OF PLURIPOTENT STEM CELLS

Pluripotent stem cells (PSCs) can become any cell of the three germ layers, which opens up the possibility to obtain insulin-secreting cells for diabetes treatment. There are two types of pluripotent cells: embryonic pluripotent stem cells (ES), derived from the inner blastocyst mass, and induced pluripotent stem cells (iPS), derived by reprogramming somatic cells into pluripotent ones. Thomson *et al *[90] were the first to report on culturing ES cells, thus marking the era of somatic cell reprogramming [91]. The differentiation potential, proliferative capacity, morphology, and gene expression profiles are similar between ES and iPS cells, which allow one to use iPS cells without the ethical restrictions associated with embryo destruction [92, 93]. Autologous human iPS cells are not cleared by immune system post-transplantation; however, there are risks associated with the rejection of implanted insulin- producing cells by the same autoimmune mechanism that leads to the emergence of diabetes.

Both ES and iPS cells can undergo differentiation *in vitro *into insulin-secreting cells [94-98]. Differentiation of PSCs into insulin-producing cells normally follows a well-defined developmental program, consisting of several stages. The first stage is endoderm formation. ES cells express multiple endoderm markers such as Sox17, Foxa2, Cxcr4; however, Sox7 is not observed [95, 96, 99-102]. Differentiation of ES and iPS cells is triggered by Nodal and Wnt signaling [95, 99, 103, 104]. The Nodal pathway is activated by activin A, a member of the TGF-β family, at a concentration of 50–100 ng/ml [105, 106]. The proportion of differentiated cells can be increased by combining activin A and certain inhibitors (wortmannin, CHIR99021 [107], sodium butyrate [96]) and activators of the Wnt-signaling pathway, such as CHIR9902 [108]. In addition, the efficacy of differentiation improves following exposure to IDE1 and IDE2 [109]. There is evidence that endodermal cells could give rise to both pancreatic and hepatic lineages. Subsequent differentiation of pancreatic cells *in vitro* requires treatment with TGF-β and BMP4 antagonists such as SU5402 and Noggin, which suppress hepatic differentiation [101].

The second stage of pancreatic differentiation is the exposure to dorsomorphine or its homolog 1 that induces the lineage commitment of Pdx1+ progenitors [96, 99, 109]. The mechanisms by which the cultured cells eventually become mature insulin-producing cells remain to be elucidated. There have been attempts to trigger differentiation *in vitro *with nicotinamide, insulin- like growth factor 1, and hepatocyte growth factor [96, 101, 103]. Further differentiation of Pdx1^+^ cells was induced with indolactam V and enhanced by retinoic acid [108]. There is evidence suggesting that the ability of PSCs to differentiate into endocrine cells strongly depends on the cell seeding density [110, 111]. Importantly, cells differentiated *in vitro *tend to produce several hormones and have an immature phenotype insensitive to the glucose level [103, 112, 113]. In this regard, progenitors are implanted to allow for a permissive *in vivo *environment for differentiation [99, 105, 114-117]. Studies in normal [99, 105] and streptozotocin-induced diabetic mice [114] demonstrate that ES cells can differentiate into functional insulin-producing cells. In addition, it is also shown that even encapsulated progenitors can be converted into mature insulin-producing cells capable of insulin secretion in diabetic mice [118].

The breakthrough work of Pagliuca *et al *[119] reports on the development of a cell differentiation protocol to produce functional insulin-secreting cells. Differentiation of human PSCs is conducted for 28–33 days in the presence of a wide set of growth factors and small molecules. The insulin-secreting cells obtained following this protocol show a glucose-responsive phenotype comparable with mature β-cells. These cells package insulin into secretory granules with an ultrastructure similar to that of adult β-cells. These cells were able to normalize the glucose level after transplantation in diabetic mice [119].

It was found that Ucn3, a corticotropin release factor, has a high expression level in β-cells and regulates glucose-dependent insulin secretion [120]. Cells differentiated* in vitro *fail to express Ucn3 [121]. At the same time, the expression levels of Ucn3 in mature and immature β-cells may differ up to 7-fold. Thus, maturation of cells *in vivo *is important to its functionality. This suggests the presence of some specific signals in the transplantation sites which trigger the differentiation and maturation of β-cells.

## DIRECT REPROGRAMMING

Reprogramming protocols developed to produce iPS cells find wide application in biomedical research. Direct reprogramming is based on the use of genetic constructs for the direction of various cell types into the desired cell type without a reversal to pluripotency. Similar to obtaining iPS cells, the direct reprogramming technique entails DNA integration (mainly through viral vectors). In particular, artificially induced Pdx1 gene expression in the liver of diabetic mice has led to the appearance of insulin+ cells near blood vessels. The conversion, however, was incomplete. Therefore, this motivated researchers to search for other genes with a synergistic effect between themselves and with Pdx1. Moreover, the search for cells suitable for programming has begun. Ductal cells show promise. In as early as 1980, Noguch *et al *[122] showed that β-cells can be derived from ductal cells. Pdx1 expression in human ductal cells activates insulin production [122]. An intraperitoneal infection of recombinant Pdx1 into streptozotocin-induced diabetic mice induced amelioration of hyperglycemia [123]. Ductal cells of adult mice transduced with an adenoviral vector carrying the Pdx1, Pax4, Ngn3, and NeuroD genes start insulin secretion [124].

According to recent research, pancreatic acinar tissue of mice can be reprogrammed through artificially induced gene expression: acinar cells first undergo differentiation into ductal cells, followed by conversion into islet cells [125]. The large number of acinar cells in the pancreas makes them an ideal model for β-cell generation studies. Acinar cells readily differentiate into insulin-producing cells when cultured *in vitro *in the presence of a low serum content supplemented with the epidermal growth factor and nicotinamide. The expression levels of glucagon, somatostatin, and pancreatic polypeptide also increase [126]. Under certain culture conditions, human acinar cells can change into duct-like structures. Dexamethasone supplementation induces an acinar-to-ductal transition, but, unfortunately, they do not differentiate into insulin-producing cells [127]. It is shown that hyperglycemia elevates the infiltration of acinar tissue by T-cells and induces differentiation of acinar cells into either β-cells or ductlike structures that can eventually become β-cells [128]. Desai *et al *reported on acianar-islet transdifferentiation in dexamethasone-treated rat pancreas [129]. Recent research suggests that acinar cells of mice can be reprogrammed by inducing expression of the Pdx1, Ngn3, and Mafa genes. The experimental mice showed a decrease in blood glucose levels, though full recovery was not observed. It is likely that the implanted cells failed to aggregate, which finally affected the cell communication regulating glucose-stimulated insulin secretion [130-132]. These results were confirmed by *in vitro *studies on a AR42J acinar cell line and then on a human exocrine cells culture [133, 134]. Importantly, Wang *et al *discovered that hyperglycemia in diabetic mice is better corrected in the case of a strong immune response elicited by the adenoviral capsid used as a vector for gene delivering [135].

The primary physiological role for α-cells is glucagon secretion, counteracting insulin by promoting glucose mobilization. The conversion of α-cells into β-cells is induced by an increase in the ectopic expression of Pax4 and Ngn3 [136]. Enforced Pdx1 expression under the Ngn3 promoter can cause α-to-β conversion during the early embryonic period; however, at later stages activation of Pdx1 has no effect on the β-cell allocation [137]. In a recent study, Chung *et al *employed pancreatic duct ligation and observed large numbers of β-cells generated from α-cells within 2 weeks [138]. Notably, the α-to-β conversion seems to occur following deep depletion of pre-existing β-cells [138, 139]. Studies involving partial elimination of β-cells failed to observe this conversion [140].

## APPLICATION OF COMMITTED CELS

The use of ES cells is ethically ambiguous, but it has other pitfalls. For example, ES- and iPS-derived transplants may generate teratomas from the residual pool of uncommitted cells. In addition, the need for immunosuppression still exists [141, 142]. Postnatal stem cells can sidestep these limitations [143-146].

Skin-derived precursors represent an available source of progenitors. They were first described by Toma *et al *[147]. They harbor broad differentiation plasticity, giving rise to multiple cell types *in vitro *(glial cells, smooth muscle cells, adipocytes). Bakhtiari *et al* [148] reported an efficient method for cryopreservation of human skin-derived precursors for long-term storage. The skin is now a promising source of autologous cells with a wide differentiation capacity and long-term storage ability [148]. Skin-derived precursors were converted* in vitro *into cells capable of glucose-dependent insulin and C-peptide secretion. The obtained cells expressed markers such as Pdx1, Nkx2.2, Pax4, NeuroD, and Isl1 found in mature β-cells [149].

The most frequently mentioned in the context of regenerative medicine postnatal stem cells are mesenchymal stem cells (MSCs) residing in various tissues [150]. They can be successfully cultured *in vitro *and readily undergo differentiation into osteogenic, adipogenic, and chondrogenic lineages using standard differentiation protocols [151]. Eyelid adipose-derived MSCs appear to be more suitable for differentiation into insulin-producing cells, since these cells originate from neural crest cells. MSCs from human periodontal ligament are also derived from neural crest cells [152-154]. However, it is currently impossible to bring MSCs close to the phenotype of β-cells under *in vitro *conditions. MSCs from umbilical cord blood offer more flexibility. Prabakar *et al *discovered that the properties of these MSCs are similar to those of ES cells, including the differentiation potential towards a pancreatic endocrine phenotype [155]. Another therapeutic option is the infusion of undifferentiated MSCs, resulting in various degrees of regeneration [153, 154, 156, 157]. Such host responses are likely to be due to the immunomodulatory, anti-inflammatory, pro-angiogenic, and trophic functions of MSCs. A more pronounced immunomodulatory effect has been described for hematopoietic stem cells that were successfully used to reset the immune system in diabetes [158, 159]. Multipotent stem cells derived from umbilical cord blood seem to be involved in the instruction of the immune system. When loaded into a circulatory device pre-seeded with umbilicalcord- blood-derived MSCs of healthy individuals, lymphocytes of type 1 diabetic patients seem to receive instructions and acquire the ability to ameliorate type 1 diabetic symptoms [160, 161].

There exists a hypothesis that an injury to the pancreas activates facultative progenitors to increase the population of β-cells. It was shown that ductal progenitors of mice can give rise to β-cells [161]. In addition, in a cohort study of chronic pancreatitis and asymptomatic fibrosis patients Gianani *et al *found that the pancreas of all patients analyzed had neogenic cells aggregated into islet-ductal structures, which appeared to be an association of the endocrine compartment with the ductal system [162]. Streptozotocin-induced diabetic mice have two types of β-cell progenitors expressing Glut2 and Pdx1/somatostatin. These cells are likely to be of ductal origin [163, 164]. Studies dedicated to investigating embryonic pancreas *in vitro *demonstrated that insulin-producing cells can originate from ductal epithelial cells. Porcine ductal cells harvested during the neonatal period can be enforced to express insulin and markers of endocrine precursors [165]. Following 3- to 4-wk incubation, human ductal cells form 3D structures which express insulin and other islets hormones. This means that they are in a state of differentiation. Moreover, insulin release in these cells is glucose-dependent [161]. Pdx1 can dramatically accelerate *in vitro* differentiation of ductal epithelial cells towards an insulin-producing phenotype [166]. In streptozotocininduced diabetic mice, it was determined that ductal cells express insulin in the early stages of inflammation, followed by termination of production [167]. This finding suggests induction of β-cell regeneration by an early-stage inflammatory response in type 1 diabetes. It is likely that new β-cells are highly prone to apoptosis. TNF-α expression induced in β-cells of mice leads to insulit rather than diabetes. This is accompanied by the development of intraislet ducts with β-cell placement, which could imply a regenerative process [168]. Similarly, transgenic mice expressing IFN-γ showed resistance to streptozotocin treatment. The transgenic mice exhibited regeneration of pancreatic duct cells and iL neogenesis [169]. Expression of Pdx1 and Msx2 in the duct cells of these mice suggests a connection between the expressed markers and ductal cells differentiation in this model [170]. In individuals with autoimmune chronic pancreatitis, T-cell mediated β-cell destruction promotes β-cell regeneration from ductal cells [171]. Type 1 diabetes patients demonstrate generation of insulin- producing Pdx1^+^ duct cells following a combined transplantation of the pancreas and a kidney.

The hyperglycemia in alloxan-induced diabetic mice can be reversed through EGF and CNTF treatment due to the generation of insulin-producing cells [172]. To elucidate the origin of the newly formed insulin- expressing cells, the authors utilized the Cre/ LoxP system to track the acinar and ductal cells. It was discovered that a total of 40% of newly formed insulinexpressing cells originated from acinar cells, whereas other cell types contributed only 4%. This allows one to suggest the existence of transdifferentiation in mammalian pancreas.

There are a number of studies that are searching for non-pancreatic sources of cells which can secrete insulin. One of the promising sites is large salivary glands. Egea *et al *identified preproinsulin I and II mRNA expression in adult rat submandibular glands [173]. Insulin in the parotid gland of rats has been found using the immunohistochemistry method [174]. It was shown that the submandibular salivary glands perform a compensatory function in diabetic mice [175]. After transplantation of the submandibular gland under the renal capsule, streptozotocin-induced diabetic mice restored normoglycemia [176]. Human and animal (mouse, rat, swine) submandibular gland cells are readily amenable for culture *in vitro *[177-179]. Under 3D culture conditions, they acquire the capacity of glucagon, albumin, or insulin expression [177, 178]. Human submandibular gland cells acquire the ability to produce C-peptide in a glucose-dependent manner in a spheroid culture system in the presence of nicotinamide [179]. Rat submandibular gland cells expressing α6β1/c-Kit maintained the morphology, proliferative capacity, and multipotency typical of stem cells for over 92 passages. The presence of activin A, exendine-4, and retinoic acid in the medium induces the expression of pancreatic markers in these cells, such as Pdx1, insulin, pancreatic polypeptide, and Ngn3 [180].

## THE USE OF BIOMATERIALS FOR THE CONSTRUCTION OF THREE-DIMENSIONAL SCAFFOLDS

It is widely known that three-dimensional culture systems can provide different advantages, compared to standard two-dimensional cultures. Cells are kept in conditions much closer to native: so, cell-cell and cell-medium interactions are promoted, and differentiation is accelerated [181]. These systems closely mimic the natural environment found *in vivo*. This stays true for pancreatic cells cultivation *in vitro *and their *in vivo* delivery.

Studies have shown that seeding cells onto a porous scaffold increases their viability and enhances the functionality of isolated iL *in vitro, *thus improving transplant outcomes. For example, rat islet cells showed an almost two-fold increase in viability and produced 4-fold more insulin when cultured on a porous polyglycolic acid scaffold as compared to 2D culturing [182]. In yet another study, a porous scaffold of poly(lactic-coglycolic acid) (PLGA) prepared by electrospinning with type I collagen-loaded pores was used. RINm5F cells were cultured on it, and insulin secretion was enhanced by 2-fold [183].

Another advantage of porous scaffolds is the opportunity to co-culture different cell types that allows one to mimic *in vivo *cell interactions. Murine islet cells cocultured with human umbilical cord endothelial cells and human prepuce fibroblasts on a PLLA\PGLA scaffold improved the survival of islet cells by up to 75%. Furthermore, the addition of fibroblasts and epithelial cells promoted the expression of Gcg, Pdx1, Nkx6.1, and Glut2 markers. The insulin secretion increased by 1.5 fold [184].

Scaffolds obviously offer a 3D structure for cell culturing; however, the fundamental role of a native extracellular matrix (ECM) on a cell’s state is increasingly being recognized. It provides not only mechanical support, but also affects cell adhesion, molecular contents, cell-to-cell interactions, and growth factors binding. Importantly, the rigidity and flexibility of ECM considerably contributes to differentiation, proliferation, viability, cell polarity, and migration [185].

The most fully characterized ECM components are laminins, a family consisting of 15–20 glycoproteins [186], each of which independently enhances insulin secretion [187]. Laminins interact with cells by binding integrins – transmembrane proteins responsible for cell adhesion and transduction of external signals to the cytoskeleton [188]. The 3D-structure of native ECM determines the topographic pattern of endocrine cells that affects secretory activity [189]. Furthermore, such components as collagens, glycoproteins, and glycosaminoglycans can independently suppress the β-cell apoptosis triggered by the loss of cell anchorage [189-195]. It was discovered that ECM components can enhance insulin secretion even in the absence of glucose [196]. ECM also has the ability to bind, store, and regulate the activity of growth factors, such as TGF-β1, which mediates the development, functioning, and regeneration of islets in the pancreas [197, 198].

**Fig. 2:**
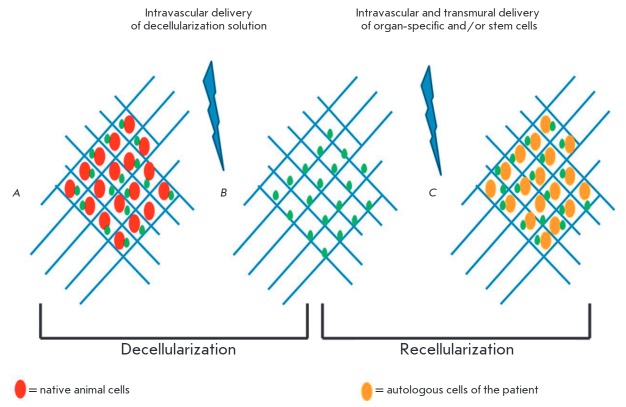
Schematic overview of decellularization-recellularization technology. A) The intact organ contains cellular components (red ellipses) and ECM (blue network), as well as growth factors (green dots); B) An acellular organ scaffold after complete removal of cellular elements; C) The organ scaffold after recellularization with autologous cells (yellow ellipses) [By 199]

Attempts are made during the development of materials for artificial 3D-scaffolds to modify their surface by coating it with molecules derived from native ECM. However, to date ECM decellularization treatment is believed to be the most promising (*Fig. 2*) [200-204]. Current approaches enable the elimination of cellular material, DNA, and surface antigens, retaining the intact structure [205]. Recent experiments have allowed researchers to obtain a porcine pancreatic extracellular matrix with preserved ECM components, including different types of collagen, elastin, fibronectin, and laminin. [206]. A decellularized membrane serves as a matrix for cell rehabitation of the organ. To date, there have been successful recellularizations of ECMs of such organs as liver [207], lungs [208], bladder [209], and mammary gland [210]. This provides hope for a positive result in the case of the pancreas as well.

## CHALLENGES AND OPPORTUNITIES

Current treatments for patients with type I diabetes are limited and do not eliminate long-term complications. Progress is evident in all lines of research connected with efforts to revive the insulin-producing function of the pancreas. Standard transplantation approaches are hampered by the shortage of donors and the risks associated with the need for immunosuppression. Those risks could be overcome by encapsulation technologies. However, there are unresolved issues in this case as well, such as poor islet longevity and a size/cell count ratio of encapsulated islet mass sufficient to provide normoglycemia without burdening the patient with discomfort. The ability of such cell types as MSCs and hematopoietic cells to address host immune responses can be very useful in preventing neogenic β-cells from repeated autoimmune ablation.

The choice of stem cells is a critical step. ES and iPS cells can differentiate towards pancreatic progenitors and/or insulin-producing cells. The use of allogenic ES cells, however, still requires immunosuppressive therapy or encapsulation. Autologous iPS cells are very costly on an individual basis and require complicated differentiation protocols. In addition, the probability of graft rejection is high due to the autoimmune response that initially leads to type 1 diabetes. The tumorigenic potential of residual undifferentiated PSCs in the implant also remains to be resolved.

Briefly, an ideal theoretical therapeutic approach would include a combined treatment: differentiated towards a pancreatic progenitor phenotype autologous iPS cells are cultured in 3D conditions in the presence of ECM components and autologous MSCs. Another possible way may be to reset the immune system with hematopoietic stem cells and to obtain new insulinproducing cells by direct reprogramming. Studies assessing the feasibility of this approach are underway to perform a thorough analysis of the potential risks associated with the biological safety and tumorigenic activity of the cells being used.

Direct reprogramming appears to be a promising method. However, data on the used protocols and the safety of this approach for obtaining insulin-producing cells is insufficient. Thus, it cannot yet proceed to the practical level.

A large number of studies have proved the positive effect of three-dimensional cell culture systems. In addition, the possibility to co-culture cells allows one to obtain a transplant which is as close to the native organ as possible. The use of decellularized ECM has shown promise; however, *in vivo *studies are in need for an understanding of the effects of the aforementioned structures on an organism.

Attempts to bring committed cells closer to the phenotype of β-cells *in vitro *have so far been unsuccessful. Overall, the current challenge in cell biology is to identify an available and accessible source of cells that are able to differentiate effectively into glucose-responsive insulin-producing cells.

## Abbreviations

iPS cells: induced pluripotent stem cells
ECM: extracellular matrix
MSCs: mesenchymal stem cells
iL: islets of Langerhans
the PSCs: pluripotent stem cells
E: embryonic development
ES cells: embryonic stem cells
